# Resistance mechanisms in collard green genotypes to *Plutella xylostella*: role of physical and morphological traits

**DOI:** 10.1002/ps.70342

**Published:** 2025-11-01

**Authors:** Aline Marques Pinheiro, Edson Luiz Lopes Baldin, Vinicius Fernandes Canassa, Alisson da Silva Santana, Thais Lohaine Braga dos Santos, André Luiz Lourenção, Carlos Gilberto Raetano

**Affiliations:** ^1^ Department of Plant Protection Faculty of Agricultural Sciences, São Paulo State University São Paulo Brazil; ^2^ West Central Research, Extension and Education Center, Department of Entomology University of Nebraska‐Lincoln North Platte NE USA; ^3^ Department of Entomology and Acarology Luiz de Queiroz College of Agriculture, University of São Paulo São Paulo Brazil

**Keywords:** antixenosis, antibiosis, waxiness, Brassicaceae, diamondback moth

## Abstract

**BACKGROUND:**

The diamondback moth, *Plutella xylostella* (Lepidoptera: Plutellidae), is one of the major pests of Brassica crops worldwide, causing significant economic losses. Using resistant plant genotypes is a promising strategy for integrated pest management, as it reduces reliance on chemical insecticides and helps mitigate resistance development. This study evaluated the resistance mechanisms of different collard green (*Brassica oleracea* var. *acephala*) genotypes to *P. xylostella*. Both antixenosis and antibiosis were investigated through oviposition preference tests and biological performance assessment.

**RESULTS:**

According to the results, there was significant variation between the genotypes. Some genotypes exhibited strong oviposition deterrence, while others affected larval survival, development time, and pupal weight. These resistance effects were associated with physical and morphological leaf traits. Genotypes 32 GUA and HS showed reduced attractiveness for oviposition and inhibited larval development and survival. These traits were linked to higher leaf wax load, increased leaf hardness, and specific leaf color parameters.

**CONCLUSION:**

These findings indicate that resistance in collard greens to *P. xylostella* is mediated by a combination of physical and morphological factors, such as plant substrate color, leaf hardness, and surface wax content. Identifying and utilizing resistant genotypes can contribute to the integrated management of *P. xylostella* and reduce the need for chemical control measures. © 2025 The Author(s). *Pest Management Science* published by John Wiley & Sons Ltd on behalf of Society of Chemical Industry.

## Graphical abstract

Resistance to *Plutella xylostella* in collard greens is mediated by antixenosis and antibiosis, associated with traits such as leaf wax, hardness, and color. Genotypes showed strong resistance potential, supporting their use in breeding and integrated pest management strategies.

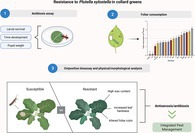

## 1 INTRODUCTION

*Plutella xylostella* (L.) (Lepidoptera: Plutellidae) is the major pest of Brassica crops worldwide.^1^, ^2^ Its larvae cause significant defoliation, leading to substantial economic losses, with management costs reaching billions of dollars.^3^, ^4^

Control of *P. xylostella* has primarily relied on synthetic insecticides. However, its short life cycle, high reproductive capacity, genetic plasticity, and frequent inappropriate use of insecticides have contributed to the rapid development of resistance.^5^ To date, over 866 cases of resistance to 101 different active ingredients have been reported worldwide.^6^ Therefore, developing strategies that can be effectively integrated into pest management programs is crucial. In this context, host plant resistance represents a valuable approach for pest control.^7^, ^8^, ^9^, ^10^

Plant resistance to insect pests is generally classified into three mechanisms: antibiosis, antixenosis, and tolerance. Antibiosis adversely affects insect biology by reducing size, weight, longevity, and causing physical deformities in insects feeding on these plants.^11^, ^12^, ^13^ Antixenosis reduces host attractiveness and disrupts insect feeding and oviposition behaviors, ultimately reducing pest populations.^14^, ^15^, ^16^, ^17^ Tolerance allow plants to maintain yield despite high infestation levels, without directly impacting insect biology or behavior.^18^, ^19^

The insect–plant interaction represent dynamic system shaped by reciprocal adaptations through coevolution.^20^ Plants have developed multiple defense mechanisms, including physical, morphological, and chemical barriers, such as leaf color that influences host selection, production of defense proteins that interfere with herbivore digestion, emission of volatile compounds that attract natural enemies, secondary metabolites with toxic or deterrent effects, high trichome density acting as mechanical barriers, epicuticular wax content that hinder insect adhesion and movement, and cell wall thickness that increases leaf toughness.^14^, ^21^, ^22^ Studies have shown that primary components of the wax layer in Brassicaceae, including triterpenoids such as amyrins and saponins, have been shown to influence host acceptance behavior in *P. xylostella* larvae.^23^, ^24^ Additionally, secondary metabolites such as glucosinolates play a key role in host recognition by Brassicaceae‐specialist insects, including the diamondback moth.^24^, ^25^, ^26^

Given the global impact of *P. xylostella* and the growing challenge of insecticide resistance, this study aimed to identify resistance mediated by antibiosis and/or antixenosis in a *Brassica oleracea* var. *acephala* germplasm bank with known genetic variability for insect resistance. Additionally, it sought to understand the associated mechanisms through the characterization of epicuticular wax content, leaf hardness, and substrate color. We hypothesized that genotypes with higher leaf hardness, wax content, and different colorimetric traits would exhibit increased resistance to *P. xylostella*, either by deterring oviposition (antixenosis) or negatively affecting larval development (antibiosis).

## 2 MATERIALS AND METHODS

### 2.1 *Plutella xylostella* rearing

The insects used in the bioassays originated from a *P. xylostella* colony maintained at the Plant Resistance and Insecticide Plants Laboratory, São Paulo State University, Brazil, under controlled conditions (25 ± 1 °C, 12 h photoperiod, and 70% ± 10% relative humidity). This colony was initially established with individuals collected from brassica crops in a conventional farming area in Pardinho, São Paulo, Brazil (23°02′28″ S, 48°22′43″ W).

Eggs and larvae were reared in 2 L plastic containers covered with organza mesh. Larvae were fed leaves of *Brassica oleracea* var. *acephala*, genotype ‘Manteiga’ (variety not evaluated in the study), obtained from glasshouse‐grown plants. Pupae and adults were kept in screened cages, where a 10% honey solution and collard greens leaves were provided for adult feeding and female oviposition, respectively. Leaves containing eggs were used to initiate new rearing cycles.

### 2.2 Obtaining collard green genotypes

Initially, 17 collard green genotypes with wide genetic variability were evaluated (Table 1). Ten genotypes were sourced from the germplasm bank of the Agronomic Institute (IAC), while the remaining genotypes were commercially obtained. Plants were grown in 2.5 L plastic pots filled with a 1:1:1 mixture of soil (dark red latosol), sand, and organic matter (cured cattle manure). For vegetative propagation, shoots were collected and placed in polystyrene trays with 128 cells filled with substrate to ensure the required number of plants per genotype. The pots were maintained in an insect‐free glasshouse and received regular irrigation, thinning, cleaning, and fertilization following recommended practices.^32^

**Table 1:** Code, names, characteristics, and history of resistance of collard green genotypes evaluated for resistance to *Plutella xylostella*

Code	Genotype^†^	Technical characteristics	History of resistance
1A	Manteiga de Ribeirão Pires I 2620	Margin irregularly sinuous, denticulate	Antixenosis to *Ascia monuste orseis* ^27^; antibiosis to *A. monuste orseis* ^28^
2B	Manteiga I 1811	Margin irregularly sinuous, denticulate	Antibiosis/antixenosis to *Brevicoryne brassicae* ^29^
5E	Gigante I 915	Green petiole, purple spots	Antibiosis/antixenosis to *Brevicoryne brassicae* ^29^
6F	Manteiga I 916	Short petiole	
8H	Manteiga de Ribeirão Pires I 2446	Green petiole	Antixenosis to *A. monuste orseis* ^27^; antibiosis to *A. monuste orseis* ^28^; antibiosis/antixenosis to *Brevicoryne brassicae* ^29^
9I	Crespa de Capão Bonito	Prominent ribs	Antibiosis to *A. monuste orseis* ^28^; antixenosis to *Bemisia tabaci* MEAM1^30^
12L	Manteiga de Jundiaí	Light green coloration	Antixenosis to *A. monuste orseis* ^27^
14N	Manteiga São José	Asymmetrical apex	Antibiosis to *Brevicoryne brassicae* ^29^
20T	Hortolândia	Orbicular limbus	Antixenosis to *Bemisia tabaci* MEAM1^30^; antixenosis to *Brevicoryne brassicae* ^29^
22V	Vale das Garças	Purple ribs	Antixenosis to *Bemisia tabaci* MEAM1^30^; antixenosis to *Brevicoryne brassicae* ^29^
32GUA	Guaranésia	Serrated edges	
34L1	Leguminosa 1	Light green coloration	Antixenosis to *Bemisia tabaci* MEAM1^30^
GAU	Gaudina	Dark green coloration, curly edges^‡^	Antibiosis to *Brevicoryne brassicae* ^29^
HI CROP	Manteiga Hi‐Crop	Intense green coloration^‡^	
HS	Manteiga HS‐20	Medium green color, smooth edges^‡^	Antixenosis to *Bemisia tabaci* MEAM1^30^
KOBE	Manteiga Kobe F1	Dark green coloration^‡^	
MGI	Manteiga ‐ Isla®	Dark green coloration^‡^	Antixenosis to *Bemisia tabaci* MEAM1^30^; antibiosis to *Brevicoryne brassicae* ^29^

^†^ Description.^31^

^‡^ Company information.

### 2.3 Antibiosis bioassay

Neonate larvae were individually maintained in Petri dishes (9.0 cm × 1.5 cm) containing leaf discs (4.6 cm^2^) from each genotype, placed on moistened filter paper (300 μL of distilled water). Each plate was considered an experimental unit, with 60 replicates per genotype, in a completely randomized design with 17 treatments (genotypes). The assay was carried out in a climate‐controlled room (26 ± 2 °C, 65% ± 10% relative humidity, 12‐h photoperiod).

Daily evaluations were performed to assess the following parameters: duration of each larval instar and the total larval phase; larval viability (%); duration of pre‐pupal and pupal phases; pupal weight (24 h post‐pupation); pupal viability (%); and the duration of development cycle (larva–adult). Pupal weights were obtained using an analytical balance (Shimadzu®, model ATY224; Shimadzu, Kyoto, Japan). In each assessment, the excrement was removed, the moistened filter paper was replaced to maintain adequate moisture for the next observation period, and the leaf discs were photographed to quantify leaf consumption.

Larval feeding behavior was assessed to characterize and differentiate resistance mechanisms (antixenosis and antibiosis). The remaining area of the leaf discs after feeding was measured using ImageJ software.^33^

### 2.4 Multiple‐choice assay

Oviposition preference was evaluated using 15 genotypes inside metal cages (3 m × 2 m × 2 m), after excluding two genotypes due to genetic material loss. Potted collard green plants at the four‐ to six‐leaf stage were randomly arranged in a circular pattern, ensuring equal spacing to prevent leaf contact. Each cage was considered one replicate, following a randomized complete block design (RCBD) with four replicates. Thirty pairs of *P. xylostella* adults (24 h old) were released at the center and on the floor of the cage, with a ratio of two pairs per genotype in the experimental setup. The oviposition was assessed over 4 days after insect release by counting the number of eggs on all leaves.

### 2.5 Wax analysis

The assay to characterize the adaxial and abaxial epicuticular wax layers of collard green leaves was performed in a completely randomized design, with 15 treatments (genotypes) and three replications. Each replication consisted of 20 leaf discs collected from the central point (middle third) of the plants. Three plants per treatment were used, totaling 60 leaf discs. Each sample was individually submerged for 20 s in a beaker (200 mL) containing 50 mL of pre‐weighed chloroform. The beakers were gently shaken during the submersion to ensure adequate wax extraction. The resulting solutions (wax + chloroform) were evaporated under a fume hood to obtain the solid wax residue. After complete evaporation, the beakers were reweighed, and the wax content was determined as a function of the mass difference.^34^

### 2.6 Leaf hardness

Hardness was measured using a CT3 Texture Analyzer (Brookfield, Middleboro, MA, USA), calibrated for a penetration depth of 3 mm at a speed of 2.0 mm s^−1^, with a TA 9/1000 probe. The results are expressed in gram‐force per centimeter (gf cm^−1^) and represent the maximum force required for the probe to penetrate collard green leaves. Evaluations followed a completely randomized design, with 15 treatments and five replicates. For each replicate, measurements were taken at ten points near the central vein on both the abaxial and adaxial surfaces of the leaves.

### 2.7 Colorimetric leaf analysis

Leaf color parameters were assessed on the adaxial and abaxial surfaces from the middle third of the plants, using reflectance in the CIE system with a Minolta Color Reader 300 colorimeter. This instrument determines L* (luminosity, ranging from black to white), a* (green intensity, ranging from red to green), and b* (yellow intensity, ranging from yellow to blue).^35^ The L* value ranges from 0 (black) to 100 (white). The value of a* is positive when the object is red and negative when the object is green. The b* value is positive when the object is yellow and negative when it is blue. The assay was carried out in a completely randomized design with 15 treatments and five replications, with each leaf considered one replication.

### 2.8 Statistical analysis

Residual normality and variance homogeneity were assessed using the Shapiro–Wilk and Levene tests, respectively. Data on developmental cycle, larval/pupal viability, pupal weight, leaf consumption, leaf hardness, wax load, and color were subjected to variance analysis, and treatment means were compared using Tukey's test (*P* < 0.05) through the LS‐Means procedure in SAS 9.2.^36^ For the number of eggs, boxplots were generated, and the 25th, 50th, and 75th percentiles were calculated for each genotype using the UNIVARIATE procedure in SAS 9.2. These percentiles were used as input variables in a *k*‐means clustering analysis performed using the FASTCLUS procedure in SAS 9.2, with the number of clusters predefined as *k* = 3. The algorithm assigned genotypes to clusters by minimizing the within‐cluster sum of squares.^10^ The analysis produced three clusters with distinct percentile profiles: cluster 1 – genotypes with high egg numbers (susceptible), cluster 2 – genotypes with intermediate egg numbers (moderately resistant), and cluster 3 – genotypes with low egg numbers (resistant). A subsequent DATA step was used to assign each genotype to its respective cluster based on the FASTCLUS output.

The oviposition preference index (OPI) was calculated using the equation: OPI = [(*T* – *P*)/(*T* + *P*)] × 100,^37^ where T represents the number of eggs on the evaluated treatment, and *P* the number of eggs on the susceptible standard genotype. The index ranges from +100 (highly stimulating) to −100 (total deterrence), with 0 indicating neutrality. The genotypes were evaluated using the chi‐square test. Negative and significant values indicated a deterrent effect, while positive and significant values indicated an oviposition‐stimulating effect.^38^ Pearson's correlation analysis was used to identify linear relationships between individual traits related to insect resistance using the Corr procedure (PROC CORR, SAS). Subsequently, principal component analysis (PCA) was applied to assess the overall multivariate structure of the data and to explore potential genotype groupings based on combined traits (insect biological variables and plants morphological variables) (R Development Core Team 2011).

## 3 RESULTS

### 3.1 Antibiosis bioassay

The mean duration of the first instar was significantly longer on larvae fed on genotypes 32GUA, HS, 12L, 22V, 6F, KOBE, and MGI compared to those fed on HI CROP (*F* = 12.39; df = 16; *P* < 0.0001; Table 2). A similar trend was observed in the second instar, with larvae fed on HS, 2B, 32GUA, HI CROP, 6F, and 1A exhibiting longer development times than those fed on genotypes 5E and KOBE (*F* = 6.25; df = 16; *P* < 0.0001). In the third instar, the longest development time were recorded in larvae fed on HS, 8H, and 1A (*F* = 6.88; df = 16; *P* < 0.0001). For the fourth instar, larvae fed on 32GUA had a significantly longer development period than those fed on KOBE (*F* = 4.91; df = 16; *P* < 0.0001).

**Table 2:** Mean number (± standard error) of first, second, third and fourth instar periods, larval period, prepupal period, pupal period and larva–adult cycle of *Plutella xylostella* in 17 collard green genotypes

Genotype	First instar	Second instar	Third instar	Fourth instar	Larval period	Prepupal period	Pupal period	Larva–adult cycle
8H	3.08 ± 0.11 bcd	2.06 ± 0.10 abcd	2.61 ± 0.09 a	2.17 ± 0.09 bc	9.89 ± 0.18 bc	1.00 ± 0.00	5.62 ± 0.10 a	16.03 ± 0.21 a
	(*n* = 60)	(*n* = 46)	(*n* = 46)	(*n* = 42)	(*n* = 39)	(*n* = 37)	(*n* = 27)	—
20T	2.66 ± 0.13 cde	1.97 ± 0.07 abcd	1.74 ± 0.13 c	2.16 ± 0.07 bc	7.80 ± 0.15 ef	1.08 ± 0.06	4.85 ± 0.08 a	16.15 ± 0.52 a
	(*n* = 60)	(*n* = 45)	(*n* = 43)	(*n* = 43)	(*n* = 36)	(*n* = 34)	(*n* = 27)	—
9I	3.05 ± 0.09 bcd	1.84 ± 0.10 bcd	1.96 ± 0.07 bc	2.06 ± 0.06 bc	8.84 ± 0.16 de	1.05 ± 0.02	5.00 ± 0.14 a	14.77 ± 0.17 ab
	(*n* = 60)	(*n* = 55)	(*n* = 52)	(*n* = 50)	(*n* = 46)	(*n* = 39)	(*n* = 27)	—
22V	3.29 ± 0.06 ab	1.85 ± 0.08 bcd	1.85 ± 0.11 bc	2.33 ± 0.08 abc	9.11 ± 0.14 cd	1.02 ± 0.02	4.14 ± 0.07 bcd	14.30 ± 0.14 ab
	(*n* = 60)	(*n* = 51)	(*n* = 49)	(*n* = 48)	(*n* = 45)	(*n* = 40)	(*n* = 33)	—
6F	3.20 ± 0.06 abc	2.13 ± 0.06 abc	1.70 ± 0.08 c	2.23 ± 0.07 bc	9.10 ± 0.12 cd	1.00 ± 0.00	4.72 ± 0.08 b	14.50 ± 0.07 ab
	(*n* = 60)	(*n* = 50)	(*n* = 36)	(*n* = 34)	(*n* = 30)	(*n* = 29)	(*n* = 22)	—
14N	2.54 ± 0.09 de	1.90 ± 0.07 bcd	1.95 ± 0.07 bc	2.21 ± 0.07 bc	8.53 ± 0.15 def	1.09 ± 0.03	4.75 ± 0.11 ab	14.37 ± 0.17 ab
	(*n* = 60)	(*n* = 50)	(*n* = 44)	(*n* = 41)	(*n* = 33)	(*n* = 33)	(*n* = 33)	—
HS	3.60 ± 0.15 ab	2.61 ± 0.11 a	2.71 ± 0.18 a	2.56 ± 0.18 ab	11.43 ± 0.26 a	1.00 ± 0.00	3.00 ± 0.15 d	15.25 ± 0.19 ab
	(*n* = 60)	(*n* = 30)	(*n* = 26)	(*n* = 21)	(*n* = 16)	(*n* = 12)	(*n* = 4)	—
1A	3.05 ± 0.14 bcd	2.13 ± 0.13 abc	2.29 ± 0.10 ab	2.17 ± 0.11 bc	8.92 ± 0.21 de	1.05 ± 0.02	4.57 ± 0.09 bc	14.21 ± 0.20 ab
	(*n* = 60)	(*n* = 56)	(*n* = 51)	(*n* = 48)	(*n* = 41)	(*n* = 40)	(*n* = 33)	—
12L	3.31 ± 0.10 ab	1.64 ± 0.06 cd	1.79 ± 0.07 bc	1.81 ± 0.08 cd	8.40 ± 0.08 def	1.04 ± 0.02	4.50 ± 0.07 bc	13.95 ± 0.11 ab
	(*n* = 60)	(*n* = 44)	(*n* = 37)	(*n* = 34)	(*n* = 27)	(*n* = 25)	(*n* = 22)	—
34L1	3.11 ± 0.12 bc	1.68 ± 0.11 cd	1.71 ± 0.07 c	2.12 ± 0.10 bc	8.26 ± 0.19 def	1.05 ± 0.04	4.71 ± 0.08 b	13.69 ± 0.14 ab
	(*n* = 60)	(*n* = 52)	(*n* = 51)	(*n* = 46)	(*n* = 41)	(*n* = 39)	(*n* = 35)	—
GAU	2.72 ± 0.08 cde	1.65 ± 0.07 cd	2.04 ± 0.07 abc	2.08 ± 0.07 bc	8.25 ± 0.08 def	1.00 ± 0.00	4.40 ± 0.13 bc	13.60 ± 0.11 abc
	(*n* = 60)	(*n* = 47)	(*n* = 41)	(*n* = 41)	(*n* = 37)	(*n* = 35)	(*n* = 28)	—
2B	2.48 ± 0.11 e	2.34 ± 0.11 a	2.10 ± 0.12 abc	2.00 ± 0.18 bcd	9.00 ± 0.32 cde	1.00 ± 0.07	3.50 ± 0.34 cd	13.66 ± 0.66 abc
	(*n* = 60)	(*n* = 52)	(*n* = 41)	(*n* = 29)	(*n* = 13)	(*n* = 9)	(*n* = 6)	—
32GUA	3.69 ± 0.11 a	2.32 ± 0.10 ab	2.00 ± 0.06 abc	3.10 ± 0.11 a	10.90 ± 0.23 ab	1.00 ± 0.00	3.50 ± 0.09 bcd	13.50 ± 0.09 abc
	(*n* = 60)	(*n* = 49)	(*n* = 37)	(*n* = 19)	(*n* = 10)	(*n* = 8)	(*n* = 2)	—
5E	2.37 ± 0.08 e	1.53 ± 0.07 d	1.81 ± 0.05 bc	2.16 ± 0.06 bc	7.77 ± 0.09 ef	1.04 ± 0.02	4.66 ± 0.66 b	13.30 ± 0.12 bc
	(*n* = 60)	(*n* = 56)	(*n* = 52)	(*n* = 49)	(*n* = 48)	(*n* = 47)	(*n* = 42)	—
HI CROP	2.27 ± 0.05 e	2.16 ± 0.08 abc	1.94 ± 0.02 bc	2.00 ± 0.06 bcd	7.92 ± 0.06 ef	1.00 ± 0.00	3.71 ± 0.06 bcd	12.57 ± 0.08 bc
	(*n* = 60)	(*n* = 54)	(*n* = 48)	(*n* = 39)	(*n* = 26)	(*n* = 20)	(*n* = 14)	
MGI	3.13 ± 0.15 abc	1.87 ± 0.12 bcd	1.65 ± 0.06 c	1.87 ± 0.06 bcd	7.84 ± 0.14 ef	1.00 ± 0.00	3.65 ± 0.07 bcd	12.50 ± 0.18 bc
	(*n* = 60)	(*n* = 45)	(*n* = 39)	(*n* = 38)	(*n* = 33)	(*n* = 27)	(*n* = 26)	—
KOBE	3.16 ± 0.06 abc	1.53 ± 0.08 d	1.79 ± 0.06 c	1.56 ± 0.06 d	7.68 ± 0.06 f	1.02 ± 0.02	3.69 ± 0.08 bcd	12.33 ± 0.12 c
	(*n* = 60)	(*n* = 49)	(*n* = 45)	(*n* = 43)	(*n* = 41)	(*n* = 39)	(*n* = 33)	—
*P*	<0.0001	<0.0001	<0.0001	<0.0001	<0.0001	0.8872	<0.0001	<0.0001

*Note*: Means followed by the same lowercase letter per column do not differ from each other by the LS‐Means adjusted by Tukey's test (*P* ≤ 0.05).

The total larval period was significantly longer in genotypes HS and 32GUA than in all other genotypes, except 8H (*F* = 18.34; df = 16; *P* < 0.0001). The duration of the prepupal phase did not differ significantly among genotypes (*F* = 0.60; df = 16; *P* = 0.8872). The pupal stage lasts longer in genotypes 8H, 9I, 20T, and 14N, compared to several other genotypes (*F* = 15.73; df = 16; *P* < 0.0001). The overall larva‐to‐adult was significantly longer in genotypes 8H and 20T than in KOBE (*F* = 12.67; df = 16; *P* < 0.0001).

**Figure 1:**
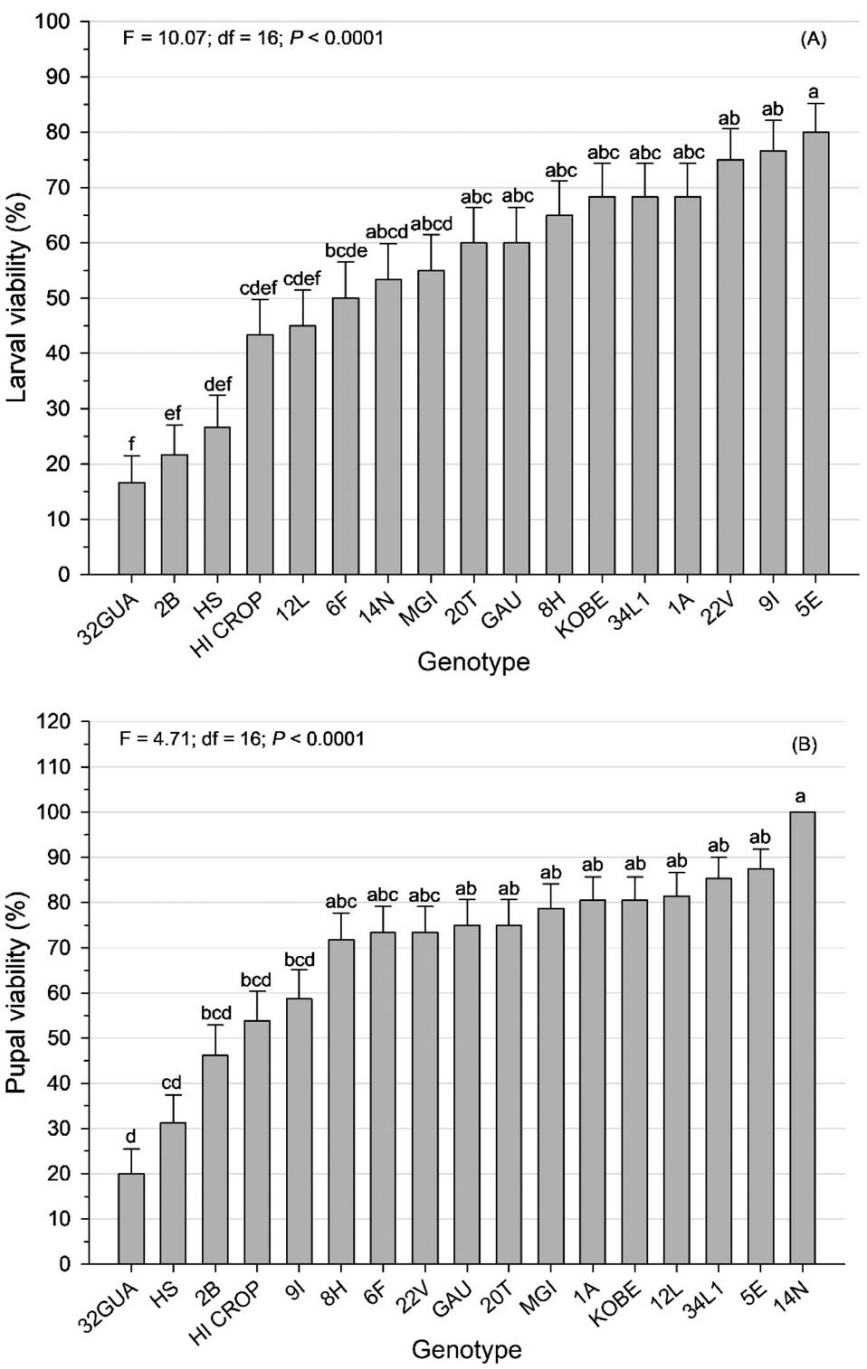
Means (± standard error) of larval viability (A) and pupal viability (B) of *Plutella xylostella* in collard green genotypes. Means followed by the same lowercase letter do not differ from each other by the LS‐Means adjusted by Tukey's test (*P* ≤ 0.05).

Larval viability on 32GUA was among the lowest observed values and did not differ from those of 2B, HS, HI CROP, and 12L, but was significantly lower than in the remaining genotypes (*F* = 10.07; df = 16; *P* < 0.0001; Fig. 1(A)). Pupal viability also differed significantly among genotypes (*F* = 4.71; df = 16; *P* < 0.0001), with 32GUA and HS resulting in the lowest survival rates (Fig. 1(B)).

Pupal weight also differed significantly among genotypes (*F* = 5.33; df = 16; *P* < 0.0001; Table 3). Pupae from larvae reared on genotype 8H had the lowest mean weight, compared to those from 5E, 6F, MGI, and KOBE.

**Table 3:** Mean weight (± standard error) of *Plutella xylostella* pupae from larvae fed different collard green genotypes

Genotype	Pupae weight (mg)
8H	3.88 ± 0.21 c
14N	4.25 ± 0.20 bc
HS	4.40 ± 0.42 abc
9I	4.45 ± 0.20 abc
2B	4.46 ± 0.44 abc
20T	4.48 ± 0.19 abc
12L	4.50 ± 0.32 abc
GAU	4.58 ± 0.22 abc
32GUA	4.97 ± 0.44 abc
1A	5.09 ± 0.21 abc
34L1	5.11 ± 0.21 abc
HI CROP	5.12 ± 0.29 abc
22V	5.21 ± 0.23 ab
5E	5.29 ± 0.19 a
6F	5.54 ± 0.24 a
MGI	5.62 ± 0.25 a
KOBE	5.77 ± 0.22 a
*P*	<0.0001

*Note*: Means followed by the same lowercase letter per column do not differ from each other by the LS‐Means adjusted by Tukey's test (*P* ≤ 0.05).

### 3.2 Foliar consumption

**Figure 2:**
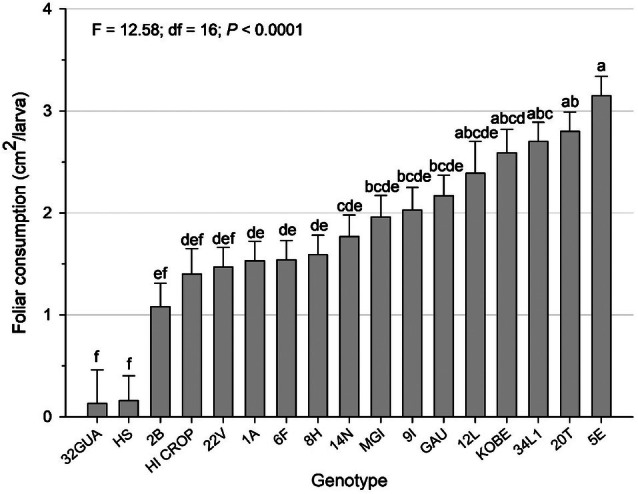
Total leaf consumption (± standard error) of *Plutella xylostella* larvae fed with leaves of collard green genotypes. Total leaf consumption considers the total consumed by the individual until its death or passage to the prepupal phase. Means followed by the same lowercase letter do not differ from each other by the LS‐Means adjusted by Tukey's test (*P* ≤ 0.05).

Leaf consumption varied significantly among genotypes, with 32GUA and HS being the least consumed, not differing only from 2B, HI CROP, and 22V (*F* = 12.58; df = 16; *P* < 0.0001; Fig. 2).

### 3.3 Multiple‐choice assay

**Figure 3:**
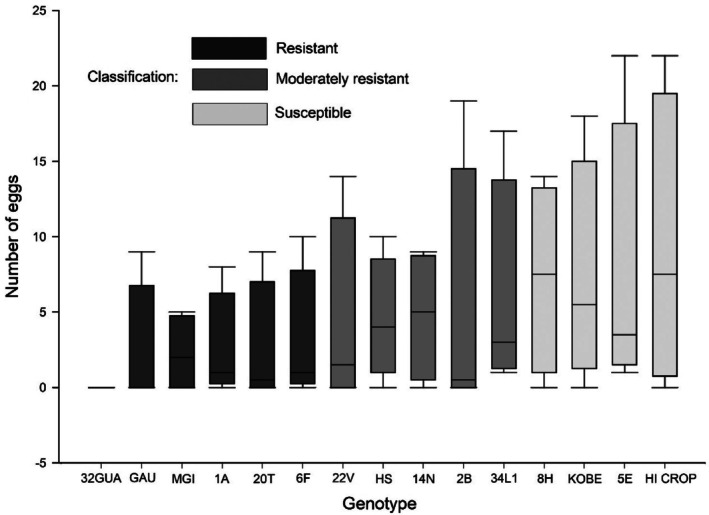
Number of *Plutella xylostella* eggs in collard green genotypes after 96 h of infestation in a free‐choice experiment conducted in a glasshouse. The solid line inside the box represents the median (50th percentile), and the lower and upper edges of the box correspond to the 25th and 75th percentiles, respectively. Genotypes were classified into three resistance categories (resistant, moderately resistant, and susceptible) based on cluster analysis.

Based on oviposition data, genotypes were clustered into three resistance categories. Genotypes 32GUA, GAU, and MGI exhibited the lowest number of eggs and were classified as strongly deterrent (Fig. 3). Genotypes 22V, HS, 14N, 2B, and 34L1 showed intermediate egg counts and were classified as moderately resistant. The remaining genotypes (8H, KOBE, 5E, and HI CROP) had the highest number of eggs and were therefore classified as susceptible.

**Figure 4:**
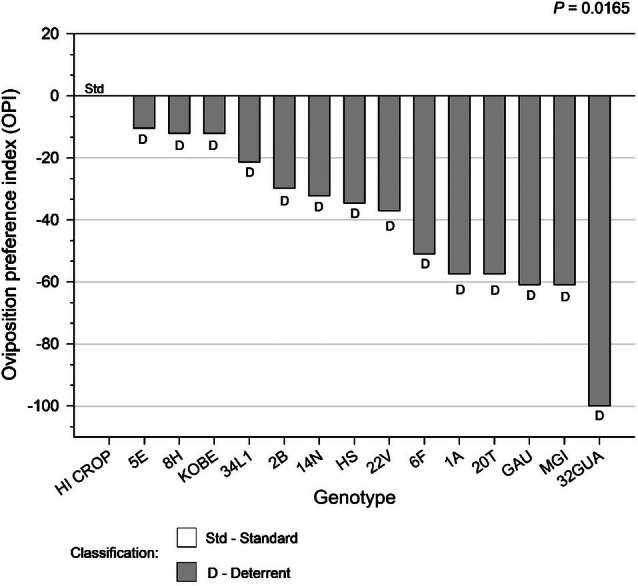
Oviposition preference index (OPI) of *Plutella xylostella* and classification of collard greens genotypes after 96 h in a free‐choice test conducted in a glasshouse, by the chi‐square test.

The OPI revealed the greatest contrast between the susceptible standard (HI CROP) and genotype 32GUA, which was classified as highly deterrent (*P* = 0.0165; Fig. 4). All other genotypes also differed significantly from HI CROP, exhibiting negative values and thus classified as deterrents in the assay.

### 3.4 Leaf hardness and wax analysis

Genotypes 22V, 2B, and 8H exhibited highest leaf hardness (*F* = 2.84; df = 14; *P* = 0.0025; Table 4). Genotype HS had the highest wax load (*F* = 3.39; df = 14; *P* = 0.0025). Due to loss of genetic material, genotypes 12L and 9I were not included in these analyses.

**Table 4:** Means (± standard error) of leaf hardness, total wax content and wax content/20 leaf discs (mg) obtained from leaves of 15 collard green genotypes

Genotype	Hardness (gf cm^2^)	Wax content (mg)
22V	0.0768 ± 0.010 a	20.00 ± 0.00 ab
2B	0.0764 ± 0.004 a	13.33 ± 3.33 ab
8H	0.0724 ± 0.004 a	20.00 ± 5.77 ab
1A	0.0672 ± 0.007 ab	10.00 ± 0.00 ab
34L1	0.0608 ± 0.010 ab	10.00 ± 5.77 ab
HS	0.0604 ± 0.004 ab	26.66 ± 3.33 a
20T	0.0592 ± 0.002 ab	13.33 ± 3.33 ab
14N	0.0584 ± 0.005 ab	13.33 ± 3.33 ab
6F	0.0576 ± 0.005 ab	6.66 ± 3.33 b
HI CROP	0.0540 ± 0.003 ab	3.33 ± 3.33 b
KOBE	0.0524 ± 0.006 ab	3.33 ± 3.33 b
GAU	0.0524 ± 0.003 ab	20.00 ± 5.77 ab
5E	0.0492 ± 0.009 ab	10.00 ± 0.00 ab
32GUA	0.0464 ± 0.006 ab	13.33 ± 3.33 ab
MGI	0.0396 ± 0.002 b	10.00 ± 0.00 ab
*P*	0.0025	0.0025

*Note*: Means followed by the same lowercase letter per column do not differ from each other by the LS‐Means adjusted by Tukey's test (*P* ≤ 0.05).

### 3.5 Colorimetric analysis

The luminosity (L*) differed significantly among genotypes on both the adaxial and abaxial leaf surfaces (*F* = 9.95; df = 14; *P* < 0.0001; Table 5). Genotype 22V exhibited the highest luminosity. The lowest green intensity (a*) values were observed in genotypes GAU and 34L1, while HI CROP, 2B, HS, 1A, and KOBE showed higher green intensity. In contrast, genotype 22V exhibited a distinct leaf coloration compared to the other genotypes, with a positive a* value indicating a reddish hue. The lowest and highest yellow intensity (b*) values were observed in genotypes 22V and 34L1, respectively.

**Table 5:** Means (± standard error) of colorimetric parameters obtained from leaves of 15 collard green genotypes

Genotype	L*^†^	a*^‡^	b*^§^
22V	44.63 ± 1.89 f	0.30 ± 1.13 a	2.95 ± 1.72 f
GAU	46.50 ± 0.60 ef	−15.72 ± 0.30 d	20.27 ± 0.89 abc
HS	51.74 ± 1.59 cdef	−10.51 ± 0.77 b	12.97 ± 0.95 cde
MGI	49.50 ± 0.93 def	−13.17 ± 0.79 bcd	13.92 ± 1.67 bcde
KOBE	50.77 ± 1.39 cdef	−10.74 ± 0.48 b	8.90 ± 1.00 ef
HI CROP	54.34 ± 1.12 bcde	−9.95 ± 0.87 b	8.80 ± 1.98 ef
2B	55.14 ± 1.11 bcd	−10.39 ± 0.35 b	12.89 ± 0.85 cde
14N	54.02 ± 1.36 bcde	−11.62 ± 0.74 bcd	12.11 ± 1.35 de
5E	55.46 ± 2.88 bcd	−12.50 ± 1.61 bcd	19.20 ± 1.11 abcd
6F	55.87 ± 0.72 bcd	−12.01 ± 0.57 bcd	17.95 ± 1.96 abcd
32GUA	58.05 ± 2.05 abc	−11.15 ± 0.93 bc	13.93 ± 1.04 bcde
8H	57.06 ± 1.54 abcd	−12.16 ± 1.11 bcd	18.53 ± 0.62 abcd
20T	57.13 ± 1.39 abcd	−15.32 ± 0.55 cd	21.68 ± 2.25 ab
34L1	59.87 ± 1.92 ab	−15.59 ± 0.94 d	22.83 ± 2.96 a
1A	64.14 ± 1.76 a	−10.54 ± 0.52 b	14.02 ± 1.12 bcde
*P*	<0.0001	<0.0001	<0.0001

*Note*: Means followed by the same lowercase letter per column do not differ from each other by the LS‐Means adjusted by the Tukey test (*P* ≤ 0.05).

^†^ Luminosity.

^‡^ Chroma, varies from red to green.

^§^ Chroma, varies from yellow to blue.

### 3.6 Correlations and multivariate analysis

Significant correlations were observed among several traits (Table 6). Total leaf consumption was negatively correlated with surface wax content (*r* = −0.38; *P* = 0.0088) and leaf hardness (*r* = −0.46; *P* < 0.0001). The number of eggs laid was negatively correlated with surface wax content (*r* = −0.42; *P* = 0.0033). Leaf hardness was positively correlated with surface wax content (*r* = 0.38; *P* = 0.0084).

**Table 6:** Pearson correlation coefficients (*r*) and probabilities (*P*) between *Plutella xylostella* parameters and characteristics of collard green genotypes

Parameter	Coefficient	Pupae weight (mg)	Hardness (gf cm^2^)	Wax (mg)	Colorimetric parameters		
					L*	a*	b*
Foliar consumption	*r*	0.47	−0.46	−0.38	—	—	—
	*P*	<0.0001	<0.0001	0.0088	—	—	—
Pupae weight (mg)	*r*	—	—	—	—	—	—
	*P*	—	—	—	—	—	—
Hardness (gf cm^2^)	*r*	—	—	0.38	—	—	—
	*P*	—	—	0.0084	—	—	—
Number of eggs	*r*	—	—	−0.42	−0.37	0.34	−0.42
	*P*	—	—	0.0033	0.0035	0.0072	0.0006

**Figure 5:**
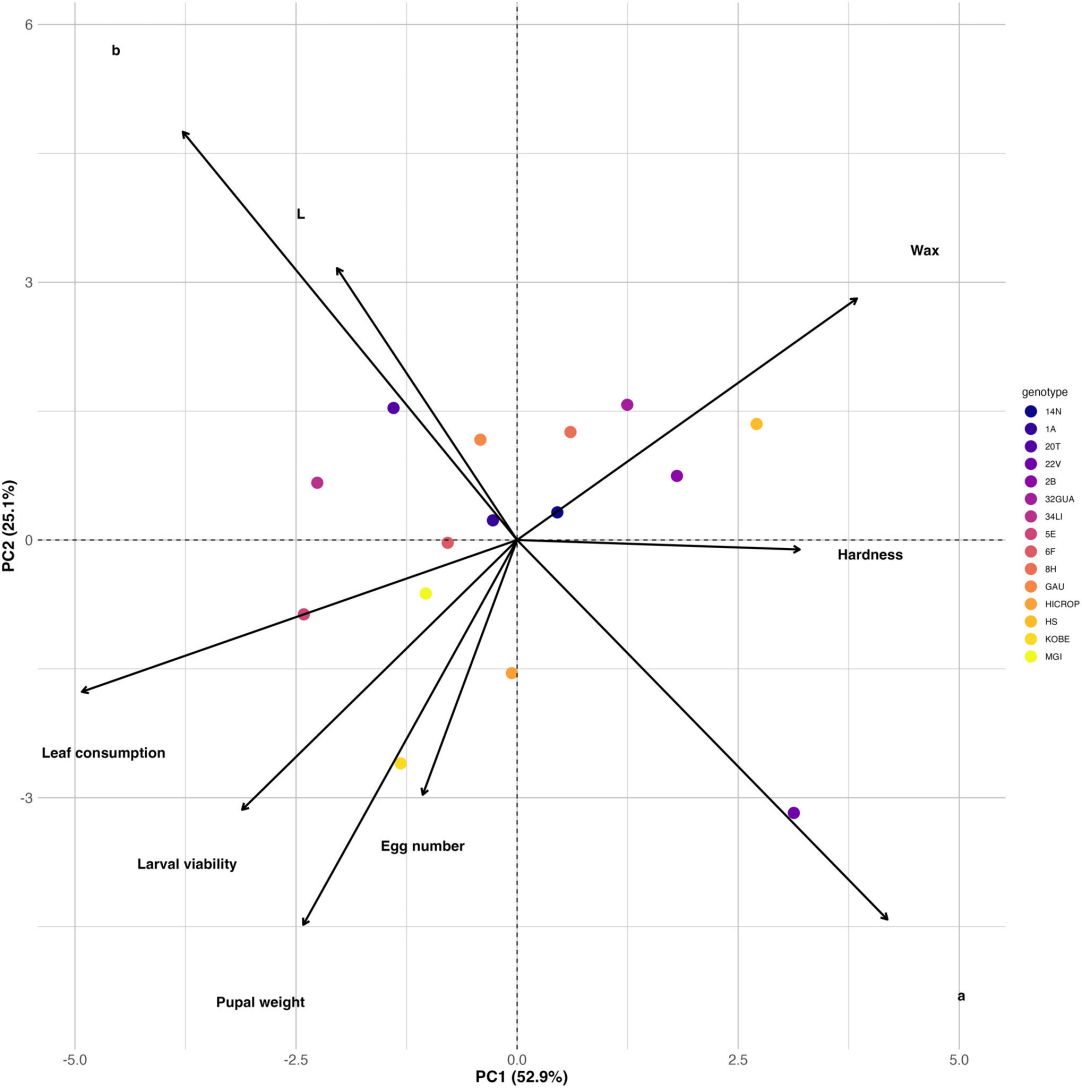
Ordination diagram of principal component analysis (PCA) based on morphological and biological traits evaluated across 15 collard green genotypes exposed to *Plutella xylostella*. The percentages of total variance represented by principal component one (PC1) (*x* axis) and principal component two (PC2) (*y* axis) are presented in parentheses. *L*: Luminosity; *a*: chroma, varies from red to green; *b*: chroma, varies from yellow to blue. The full names of the genotypes can be found in Table 1.

PCA revealed that the first two principal components (PC1 and PC2) accounted for 55.7% of the total variance, with 31.0% explained by PC1 and 24.7% by PC2 (Fig. 5). PC1 was primarily associated with leaf consumption (loading = −0.49), color parameter a* (0.41), wax load (0.38), and color parameter b* (−0.37). PC2 was mainly influenced by pupal weight (−0.44), and b* (0.47). The biplot showed distinct clustering among genotypes, with 32 GUA and HS positioned distant from the origin, indicating that these genotypes exhibit unique combinations of morphological traits, which affect the biological parameters of the insect. Genotype 32 GUA had high scores on PC2 and low scores on PC3, which were associated with reduced oviposition and increased pupal weight. In contrast genotype HS exhibited high positive scores on PC1, associated with reduced leaf consumption. Genotypes HI CROP and MGI clustered near the origin or in opposite quadrants.

## 4 DISCUSSION

In this study, we evaluated the adverse effects of diverse *Brassica oleracea* var. *acephala* genotypes on key biological parameters of *P. xylostella*. Our results demonstrate that the genotypes affected larval duration, total development time, viability, pupal weight, and leaf consumption.

The prolongation of the immature stage may result from ingestion of toxic compounds typically found in genotypes exhibiting antibiosis, which can inhibit insect development.^39^ However, this effect can also occur in genotypes with high levels of antixenosis, which may impair insect feeding due to poor nutritional properties or the overall absence of feeding stimulants.^40^ These changes in the immature stages and overall life cycle are relevant for pest management, as *P. xylostella* is characterized by a short life cycle and a high number of generations per year, which facilitates the selection and spread of resistance alleles already present in the population.^5^ Therefore, a host plant that prolongs insect development time may contribute to slowing resistance development, provided the delay is sufficient to reduce the number of pest generations per year.^41^

High mortality was observed in the immature stages of *P. xylostella* when fed on genotypes 32 GUA, 2B, and HS. Additionally, genotype 8H significantly reduced pupal weight. Plant defense compounds can disrupt insect development by increasing larval development time, reducing pupal weight and viability, and decreasing reproductive capacity.^22^, ^42^, ^43^ Glucosinolates, which are the primary and secondary metabolites in Brassicaceae plants, play a crucial role in defense against insect herbivores and pathogens.^44^, ^45^, ^46^ Previous studies have reported high concentrations of the glucosinolates glucobrassicin and gluconapin in genotype HS,^34^ which may reduce *P. xylostella* feeding and viability. Lower pupal weight may be associated with low nutrient reserves accumulated during the larval stage, likely due to the lower nutritional value of the host plant, as observed in diets with imbalanced protein‐to‐carbohydrate ratio.^47^ As expected, a positive correlation was observed between leaf consumption rate and pupal weight, indicating that larvae with lower foliar intake developed into lighter pupae, as seen in genotype 8H. This finding suggests that the host plant may be deficient in proteins.^47^ Adverse effects on development time and pupal weight can negatively impact insect reproductive performance, reducing mating frequency, oviposition, and fertility.^48^, ^49^ However, when a host plant is low in nitrogen, herbivorous insects often increase leaf consumption to compensate for the lower nutritional quality and meet their protein requirements.^50^, ^51^

Low leaf consumption observed in genotypes 32 GUA and HS suggests antixenosis. Feeding deterrence may result from chemical cues or morphological barriers such as trichomes or epicuticular wax.^14^, ^21^ Indeed, a negative correlation between leaf consumption and wax content, as well as between the wax content and oviposition, was found in this study. The role of wax load in insect resistance appears to be complex. Although it has been proposed that *P. xylostella* larvae prefer wild‐type genotypes with higher wax levels compared to glossy mutants,^52^ other studies have associated waxy plants with reduced feeding, survival, or oviposition^53^, ^54^ The epicuticular wax forms an important chemical barrier to insect–plant interactions. In *P. xylostella*, oviposition preference may be negatively influenced by an increased amount of surface wax, as crystalline epicuticular waxes can hinder egg adhesion, alter the perception of volatile compounds, and modify the microenvironmental conditions of the leaf surface.^55^, ^56^ Leaf waxes in Brassica species are mainly composed of lipophilic compounds like alkenes, paraffins, and saponins. Although glucosinolates are not wax constituents, they may be present on the leaf surface and play a role in plant–insect interactions^57^, ^58^ While *P. xylostella* has developed adaptive mechanisms to overcome the effects of glucosinolates,^59^, ^60^ the saponins present in leaf wax act as feeding inhibitors for its larvae.^24^ This may partially explain the low leaf consumption observed in genotype HS, which exhibited the highest wax load. However, the specific composition of epicuticular waxes, such as the relative abundance of compounds like amyrins, alkanes, alkenes, and long‐chain alcohols, likely plays a more critical role in influencing insect feeding behavior, but no chemical characterization was performed, and the specific compounds responsible for this effect remain unknown.

In this study, wax content was positively correlated with leaf hardness, while leaf consumption was negatively associated with hardness. This morphological trait may have acted as a feeding constraint and a mortality factor for the larval instars of *P. xylostella*, as harder leaves can cause mechanical damage to herbivores.^61^ Moreover, these morphological attributes are particularly relevant because *Brassica oleracea* plants with higher wax content and hardness have a lower abundance of insect pests, including *P. xylostella*.^62^ A significant positive correlation was also observed between leaf greenness of collard green genotypes and the number of eggs laid by *P. xylostella*. This result suggests that genotypes with greener leaves were more attractive for oviposition, highlighting the importance of visual cues in the host selection process.^63^ More intense leaf coloration may act as a visual cue for host selection. One possible explanation is that greener leaves are often associated with higher nitrogen levels and greater nutritional quality for larval development,^64^ although nitrogen content was not measured in this study. Furthermore, the negative correlation observed between the luminosity value (L*) and *P. xylostella* oviposition suggests that genotypes with medium to dark green leaves (GAU, HI CROP, HS, KOBE, and MGI) were more preferred by females. In addition, the lack of preference for leaves with a more yellowish color (higher b* values) may reflect the females' ability to avoid less nutritious substrates. Thus, the negative trend between b* and the number of eggs reinforces the role of color as an indicator of host quality.^20^, ^65^ Similar results were reported, highlighting the relevance of color parameters of *Brassica oleracea* var. *acephala* genotypes in the oviposition decision‐making of *Ascia monuste orseis*.^27^

The PCA provided an integrated view of the relationships between insect biological responses and morphological traits of collard green genotypes. PC1, which explained most of the variance, was negatively correlated with leaf consumption and positively associated with wax load and the a* color parameter (reddish tones). Genotypes with high scores on PC1, such as HS, exhibited lower leaf consumption, suggesting antixenosis potentially mediated by surface wax and color parameter. In contrast, PC2 was negatively associated with pupal weight and positively with the b* parameter (yellow hue), indicating that genotypes with more yellowish leaves tended to reduce larval performance, possibly due to lower nutritional quality or increased presence of defensive compounds. These results support the role of foliar color as a proxy for nutritional or chemical factors influencing resistance.

Genotype 32 GUA presented high scores on PC2 and low scores on PC3. PC3 was mainly associated with egg number, larval viability, and leaf consumption (positive loadings), and negatively with pupal weight. Therefore, 32 GUA's position in this axis suggest reduced oviposition and foliar intake, along with higher pupal weight, indicating an antixenosis profile without apparent antibiosis. Although females avoided this genotype, larvae that developed on it achieved better growth, reinforcing the notion that antixenosis and antibiosis may act independently. Conversely, HI CROP and MGI scored near the origin of the biplot, indicating weak expression of resistance‐related traits across the evaluated variables. This finding reinforces the suitability of HI CROP as a susceptible standard genotype, especially given its consistent performance across bioassays.

Previous studies have reported resistance of genotypes HS, 8H, 9I, and 22V to other pest species, including antixenosis in genotypes HS, 9I, and 22V to *Bemisia tabaci* MEAM1,^30^ antixenosis in genotype 9I and antixenosis/antibiosis in genotype 8H against *A. monuste orseis*,^27^, ^28^ and antibiosis/antixenosis in genotype 8 H and antixenosis in genotype 22 V to *Brevicoryne brassicae*.^29^ These findings reinforce the importance of employing these cultivars in an integrated pest management (IPM) program for the pest complex that affects *Brassica oleracea* var. *acephala*. Resistant plants remain the best way to manage multiple insect pests while ensuring sustainable food production,^66^ particularly due to their persistence, ease of deployment, specificity, cumulative effects, low cost, compatibility with other IPM methods, and reduced environmental and human health risks.^67^ However, identifying and incorporating resistance within a single genotype remains a major challenge, despite previous research.^68^, ^69^, ^70^ This is because resistance to one species may sometimes be associated with susceptibility to another, or even with deleterious effects on agronomic traits.

## 5 CONCLUSION

This study identified significant variation in resistance levels to *P. xylostella* among collard green genotypes, with indications of both antixenosis and antibiosis. Genotypes such as 32 GUA and HS consistently reduced oviposition and negatively affected larval development and survival. These resistance responses were associated with higher leaf wax content, increased hardness, and different foliar color parameters. However, it is important to emphasize that these findings represent possible associations rather than definitive causal mechanisms. Further research is needed to confirm the functional roles of these morphological traits in mediating resistance. Although resistant genotypes showed promising traits under controlled conditions, their effective integration into IPM programs should be considered with caution. Field‐based studies and assessments of agronomic performance and trade‐offs, including potential susceptibility to other pests, are needed before practical recommendations can be made.

## CONFLICT OF INTEREST

All authors declare no conflict of interest.

## Data Availability

The data that support the findings of this study are available from the corresponding author upon reasonable request.
